# The quantitative genetics of fitness in a wild seabird

**DOI:** 10.1111/evo.14516

**Published:** 2022-06-15

**Authors:** Maria Moiron, Anne Charmantier, Sandra Bouwhuis

**Affiliations:** ^1^ Centre d'Ecologie Fonctionnelle et Evolutive Univ Montpellier, CNRS, EPHE, IRD Montpellier France; ^2^ Institute of Avian Research An der Vogelwarte 21 26386 Wilhelmshaven Germany

**Keywords:** adaptive potential, additive genetic variance, heritability, lifetime reproductive success, log‐normal fitness

## Abstract

Additive genetic variance in fitness is a prerequisite for adaptive evolution, as a trait must be genetically correlated with fitness to evolve. Despite its relevance, additive genetic variance in fitness has not often been estimated in nature. Here, we investigate additive genetic variance in lifetime and annual fitness components in common terns (*Sterna hirundo*). Using 28 years of data comprising approximately 6000 pedigreed individuals, we find that additive genetic variances in the zero‐inflated and Poisson components of lifetime fitness were effectively zero but estimated with high uncertainty. Similarly, additive genetic variances in adult annual reproductive success and survival did not differ from zero but were again associated with high uncertainty. Simulations suggested that we would be able to detect additive genetic variances as low as 0.05 for the zero‐inflated component of fitness but not for the Poisson component, for which adequate statistical power would require approximately two more decades (four tern generations) of data collection. As such, our study suggests heritable variance in common tern fitness to be rather low if not zero, shows how studying the quantitative genetics of fitness in natural populations remains challenging, and highlights the importance of maintaining long‐term individual‐based studies of natural populations.

Fisher's Fundamental Theorem of Natural Selection postulates that “the rate of increase in fitness of any organism at any time is equal to its genetic variance in fitness at that time” (Fisher 1930). As such, additive genetic variance in fitness, equivalent to the change in mean fitness resulting from selection, has been considered the single most useful statistic quantifying selection (Burt 1995). Genetic variation in fitness is also a prerequisite for adaptive evolution, as a trait must be genetically correlated with fitness to evolve through natural selection (Robertson 1966; Price 1970). Hence, understanding the quantitative genetics of individual variation in fitness is arguably one of the most important aims in evolutionary ecology (Burt 1995; Ellegren and Sheldon 2008; Walsh and Blows 2009; Gomulkiewicz and Shaw 2013; Shaw and Shaw 2014; Hendry et al. 2018).

Considerable debate has surrounded the question of whether additive genetic variation in fitness is expected to be low (e.g., Jones 1987; Burt 1995; Houle et al. 1996; Merilä and Sheldon 1999; Shaw and Shaw 2014), particularly under which conditions (e.g., Cheverud and Routman 1995; Whitlock et al. 1995). Empirical estimates of additive genetic variance in fitness from wild populations are relatively scarce (e.g., Gustafsson 1986; Kruuk et al. 2000; Merilä and Sheldon 2000; McCleery et al. 2004; Coltman et al. 2005; Brommer et al. 2007; Foerster et al. 2007; Teplitsky et al. 2009; Wheelwright et al. 2014; McFarlane et al. 2014, McFarlane et al. 2015; Wolak et al. 2018; de Villemereuil et al. 2019) and have thus far not shed much light on this debate, since estimates vary substantially, with many estimates close to zero and few large estimates (review by Hendry et al. 2018). Overall, Hendry et al. (2018) tentatively concluded that the evolvability of fitness (measured as the square of the coefficient of additive genetic variance in fitness) is usually less than 0.2.

Data constraints might partially explain the paucity of studies testing for the heritability of fitness in the wild and the heterogeneity among estimates of additive genetic variance, although steadily growing datasets collected from long‐term study populations gradually alleviate the problem (Clutton‐Brock and Sheldon 2010). This increased data availability was recently accompanied by the development of (i) statistical tools designed to deal with the non‐Gaussian distributions that often characterize fitness data (de Villemereuil et al. 2016; de Villemereuil 2018), as well as (ii) theoretical frameworks that facilitate the evolutionary inference of quantitative genetic parameters based on these data distributions (Morrissey and Bonnet 2019). To date, only four studies have modeled the quantitative genetics of fitness in wild populations assuming a non‐Gaussian distribution (McFarlane et al. 2014; McFarlane et al. 2015; Wolak et al. 2018; de Villemereuil et al. 2019). Additive genetic variance in fitness was estimated to be very small in North American red squirrels (*Tamiasciurus hudsonicus*) (V_A_ ∼ 0, 95% = 5.2×10^−07^ ‐ 1.1, McFarlane et al. 2014, see also McFarlane et al. 2015). In birds, de Villemereuil et al. (2019) showed that hihis (*Notiomystis cincta*) in New Zealand had negligible additive genetic variance in lifetime fitness (V_A zero‐Inflated component_ ∼ 0, 95% CI = 1.4×10^−11^ ‐ 0.0038 and V_A Poisson component_ = 0.0078, 95% CI = 2.3×10^−10^ ‐ 5.7), while Wolak et al. (2018) found that the song sparrows (*Melospiza melodia*) of Mandarte Island in Canada harbored substantial additive genetic variance in female and male fitness (V_A female_ = 2.01, 95% CI = 0.21 – 3.93; V_A male_ = 1.72, 95% CI = 0.27 – 3.39).

Here, we present phenotypic and pedigree data obtained from a 28‐year individual‐based study on common terns (*Sterna hirundo*). The common tern is a Nearctic and Palearctic colonially breeding, serially monogamous and migratory seabird. The study colony is located in northern Germany; common terns from this colony spend their winters in western Africa and return to the breeding colony in early spring to breed or prospect potential breeding locations (Becker and Ludwigs 2004). Common terns breed annually, both parents incubate and feed the chicks, and extrapair paternity is rare (González‐Solís et al. 2001; Becker and Ludwigs 2004). Applying a series of “animal models” to data from almost 6000 pedigreed individuals across five generations, we investigate additive genetic variance for lifetime fitness (assessed as the total number of fledglings produced by a locally born fledgling) and two of its underlying annual components: annual reproductive success and adult annual survival.

## Methods

### STUDY SYSTEM

Fitness and pedigree data were collected between 1992 and 2019 as part of a long‐term study of a common tern population located at the Banter See on the German North Sea coast (53°36´N, 08°06´E). The Banter See colony consists of six concrete islands, each of which is surrounded by a 60‐cm wall. Walls are equipped with 44 elevated platforms, each containing an antenna that reads transponder codes. The individual‐based study at the Banter See was initiated in 1992, when 101 adult birds were caught and marked with individually numbered subcutaneously injected transponders. Since 1992, all locally hatched birds have been similarly marked with a transponder shortly before fledging, and the presence and reproductive performance of marked individuals have been monitored following a standard protocol (Becker and Wendeln 1997). As part of this protocol, the colony is checked for new clutches every 2−3 days throughout the breeding season (Zhang et al. 2015). Parents are identified using portable antennae placed around each nest for 1−2 days during incubation, which are shared by both partners. Pairs could rear up to three chicks per brood (mean successful brood size 0.41 ± 0.65 SD chicks) and can produce replacement clutches after the loss of eggs or chicks. Second clutches are extremely rare (Becker and Zhang 2011).

### FITNESS DATA

Our initial data selection included individuals that fledged between 1992 and 2016 because previous work showed that 97% of fledglings, if they returned, did so within the first 3 years (Vedder and Bouwhuis 2018). Since there is little standardized monitoring in areas around the focal colony, we cannot directly quantify juvenile dispersal. However, we know that there is (i) a relatively high local return rate (26% of chicks fledged between 1992 and 2016 returned to the colony, of which 14% recruited) and (ii) only rare reporting of external recruits (between 1992 and 2016, 32 fledglings from the Banter See were observed a total of 105 times in other European breeding colonies). In addition, although we cannot directly observe an individual's death, we can reliably assume it, because adult breeders at the Banter See are highly site‐faithful, evidenced by a resighting probability of breeding individuals close to one (Szostek and Becker 2012), and 96% of breeders not skipping recording by the antenna system for two or more consecutive years after first reproduction (Bouwhuis et al. 2015; Zhang et al. 2015). Based on this knowledge, we removed all birds that were observed in 2018 and/or 2019 *and* were younger than 11 years old because (i) they are known to not be, or cannot yet be assumed to be, dead, and (ii) lifetime fitness of individuals older than 10 years and those who died showed a high correlation (r > 0.8) in our dataset. Hence, we included birds that completed their life histories (n = 5836), as well as birds that were still alive but older than 10 years (n = 163), to avoid introducing a cohort truncation bias by nonrandomly removing longer‐lived birds (Hadfield 2008; Morrissey et al. 2012). To control for any potential confounding effect, we modeled whether an individual was considered dead or alive as a fixed effect (see below).

**Figure 1:**
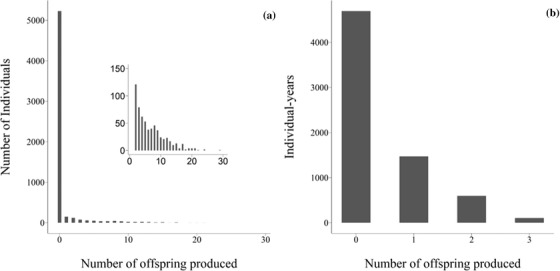
Phenotypic distributions of (a) lifetime fitness measured as the total number of fledglings a locally hatched fledgling produced in its lifetime (with the inset showing the distribution for nonzero fitness in more detail) and (b) annual reproductive success, measured as the number of fledglings an adult breeder produced in a year.

We quantified lifetime fitness as the number of local fledglings that a locally hatched fledgling produced during its lifetime for a total of 5999 locally hatched fledglings (Fig. 1a) and decomposed it into two major components: juvenile survival and adult lifetime reproductive success. Juvenile survival captures survival from fledgling to age 1, inferred from whether a fledgling became a local recruit in later years, whereas adult lifetime reproductive success captures adult survival and reproductive success from age 1 onward. These two fitness components correspond to the two mechanisms captured by the zero‐inflated Poisson distribution of lifetime fitness. We further decomposed adult lifetime reproductive success into its two components: annual reproductive success (ARS) and adult annual survival (AAS). ARS was measured as the number of fledglings that an individual produced each year between age 1 and last registration, assigning zeroes for years of skipped reproduction or registration and for years prior to recruitment (Fig. 1b). Similarly, AAS was adult survival (1/0) to the following breeding season, measured every year from age 1 to last registration (inferring missing direct observations prior to recruitment from later observations). In total, our data comprised 836 individuals with 6873 observations for ARS and AAS.

### PEDIGREE

The pedigree was constructed by assigning all fledged offspring to their social parents and then pruned to remove individuals who were either not phenotyped or not ancestors to phenotyped individuals. For the purpose of this study, the pruned pedigree comprised 6290 records. The maximum depth was five generations, and the numbers of paternities and maternities were 2417 and 2520, respectively. The numbers of full, paternal and maternal siblings were 2594, 10,229, and 9807, respectively (see Supporting Information for further information on the population relatedness structure). This social pedigree is a good approximation of the genetic pedigree because common terns exhibit very low levels of extrapair paternity (González‐Solís et al. 2001).

### QUANTITATIVE GENETIC MODELS

We applied an animal model approach that combines phenotypic information on individual fitness components with information from the social pedigree (Kruuk 2004). As such, we fitted a series of univariate animal models where fitness, or one of its components, was the response variable.

To model lifetime fitness, we fitted a univariate animal model with a zero‐inflated Poisson error distribution. We fitted a zero‐inflated Poisson distribution to better capture the nature of our metric of lifetime fitness. Zero‐inflation is often the result of a process that determines whether an event occurs or not, which differs from the Poisson process that determines how many times an event occurs. In this case, a zero‐inflated Poisson model can explicitly model the two different processes, as opposed to a Poisson model that assumes only a single process to be generating the data (Korner‐Nievergelt et al. 2015). We fitted random intercepts for individual identity linked to the pairwise relatedness matrix and for hatch‐year (to account for cohort effects; e.g., Vedder and Bouwhuis 2018). Because we modeled lifetime fitness with a zero‐inflated overdispersed Poisson distribution, we could estimate the covariance between the zero‐inflated and Poisson components for each variance component. However, a model including additive genetic and hatch‐year covariances between the zero‐inflated and Poisson components of the trait did not provide a better fit to the data; hence, we did not model such covariances. The main models presented also did not control for shared environmental effects between siblings (maternal, paternal, or brood effects) because we did not have information on parental identity for all individuals (maternal identities = 2382 and paternal identities = 2481; 1271 individuals have both maternal and paternal identities known, see Supporting Information for detailed information on the population relatedness structure) and because most fledglings came from broods where only a single individual had successfully fledged (3027 broods fledged one chick, 1145 broods two,226 broods 3, while 4 individuals could not be assigned to a brood). However, we did explore the potential effects of a shared environment (due to maternal, paternal effects, or brood effects) by running two additional animal models that included one or two shared environmental effects as random effect(s). We found that there was no major influence on our estimate of additive genetic variance in lifetime fitness components, as expected given that the model presented in the main text returned a very low (close to or zero) estimate of additive genetic variance (see Suppl. Material, Tables S1 and S2).

As fixed effects, we modeled the trait intercept and whether the individual was alive or dead at the end of the study period (categorical variable with two levels). Additionally, we performed data simulations to investigate (i) whether we can effectively detect “small but substantial” additive genetic variances in fitness (*sensu* de Villemereuil et al. 2019) given our data and pedigree structure and (ii) the improvement of our statistical power to detect small additive genetic variances in both components of lifetime fitness when the dataset and pedigree would increase in size and depth (Supporting Information, Figs. S1‐S5).

To model ARS, we assumed a Poisson error distribution with a log link function and checked whether the trait was underdispersed, which was not the case. We fitted random intercepts for individual identity linked to the pairwise relatedness matrix, individual identity not linked to the pedigree (to account for permanent environmental effects) and year of observation (to account for temporal variation across years). As fixed effects, we modeled the trait intercept and age (continuous trait ranging from 1 to 23 years), as fledgling production is known to linearly increase with age (Zhang et al. 2015) (but see Supporting Information, Table S3, for results of the same animal model without age effects).

To model AAS, we assumed a binary error distribution with a logit link function and fixed the residual variance to one. We fitted random intercepts for individual identity linked to the pairwise relatedness matrix, individual identity not linked to the pedigree (to account for permanent environmental effects) and year of observation (to account for temporal variation across years). As fixed effects, we modeled the trait intercept and age (continuous trait ranging from 1 to 23 years), as AAS is known to linearly decrease with age (Zhang et al. 2015; Vedder et al. 2021) (but see Supporting Information, Table S3, for results of the same animal model without age effects).

All quantitative genetic models were fitted using a Bayesian framework implemented in the statistical software R (v. 3.6.1, R Core Team 2019) using the R packages *MCMCglmm* (Hadfield 2010) and *QGglmm* (de Villemereuil et al. 2016). Posterior distributions were plotted using the R package *wolakR (github.com/matthewwolak/wolakR)*. Narrow‐sense heritabilities (h^2^) were conditional on the variance explained by fixed effects and were estimated as the proportion of the total phenotypic variance explained by the additive genetic variance. Evolvabilities (I_A_) were estimated by dividing the additive genetic variance by the squared population mean (Houle 1992; Hansen et al. 2011).

For all models, we used parameter‐expanded priors (Hadfield 2010). We fitted different priors for each fitness component (see Supporting Information). The number of iterations and thinning intervals were chosen for each model to ensure that the minimum MCMC effective sample size for all parameters was 1000. Burn‐in was set to a minimum of 5000 iterations. The retained effective sample sizes yielded absolute autocorrelation values <0.1 and satisfied convergence criteria based on the Heidelberger and Welch convergence diagnostic (Heidelberger and Welch 1981). We drew inferences from the posterior mode and 95% credible intervals (95% CI). To facilitate evolutionary inference (Bonnet et al. 2019; Morrissey and Bonnet 2019), we back‐transformed the latent‐scale posterior distributions of the quantitative genetic parameters to the data‐scale (de Villemereuil et al. 2016).

## Results

### QUANTITATIVE GENETICS OF LIFETIME FITNESS COMPONENTS

Among the 5999 common tern chicks that fledged between 1992 and 2016, lifetime fitness ranged between 0 and 29 fledglings (Fig. 1a). A total of 5231 (87.19%) fledglings obtained zero fitness, such that the distribution of fitness was strongly zero‐inflated (Fig. 1a).

The raw mean fitness was 0.72 ± 2.52 SD fledglings. Although this value would indicate the population to be in overall decline (a mean lifetime breeding success of two fledglings would be required for the population to be stable), population size actually varied dramatically across years and did not decline (Fig. S6), partially because there was a substantial influx of nonlocally hatched breeders that immigrated into the population (ca. 74 % ± 1 SD breeders were estimated to be immigrants in any given year between 1992 and 2020). Since we do not capture or mark immigrants, we can quantify the proportion of immigrants present in our colony in a given year, but we cannot include them in the pedigree or our individual‐based models.

Simulations showed that, given our data structure and pedigree, we would not be able to detect what might be considered a *small but substantial* signal for the zero‐inflated component of lifetime fitness: we generated a zero‐inflated component of fitness with an additive genetic variance of 0.01 and found that the average posterior mode was similar to the simulated value of V_A_ (average = 0.012 across the 100 replicates, Fig. S1), but the lower 95% CI limit was on average zero across replicates (95% CI = 0 – 0.023 and lower 95% CI exceeded a value of 0.0001 only 72 times across the 100 replicates, Fig. S1). When we simulated larger values of additive genetic variance (i.e., V_A_ = 0.05 or 0.10), our simulations showed that we would be able to detect those values (average = 0.053 and 95% CI = 0.028 – 0.083 across the 100 replicates for a simulated value of 0.05; and average = 0.102 and 95% CI = 0.064 – 0.145 for a simulated value of 0.10). The lower 95% CI always exceeded a value of 0.0001 in both simulated cases (Figs. S3 and S4).

**Figure 2:**
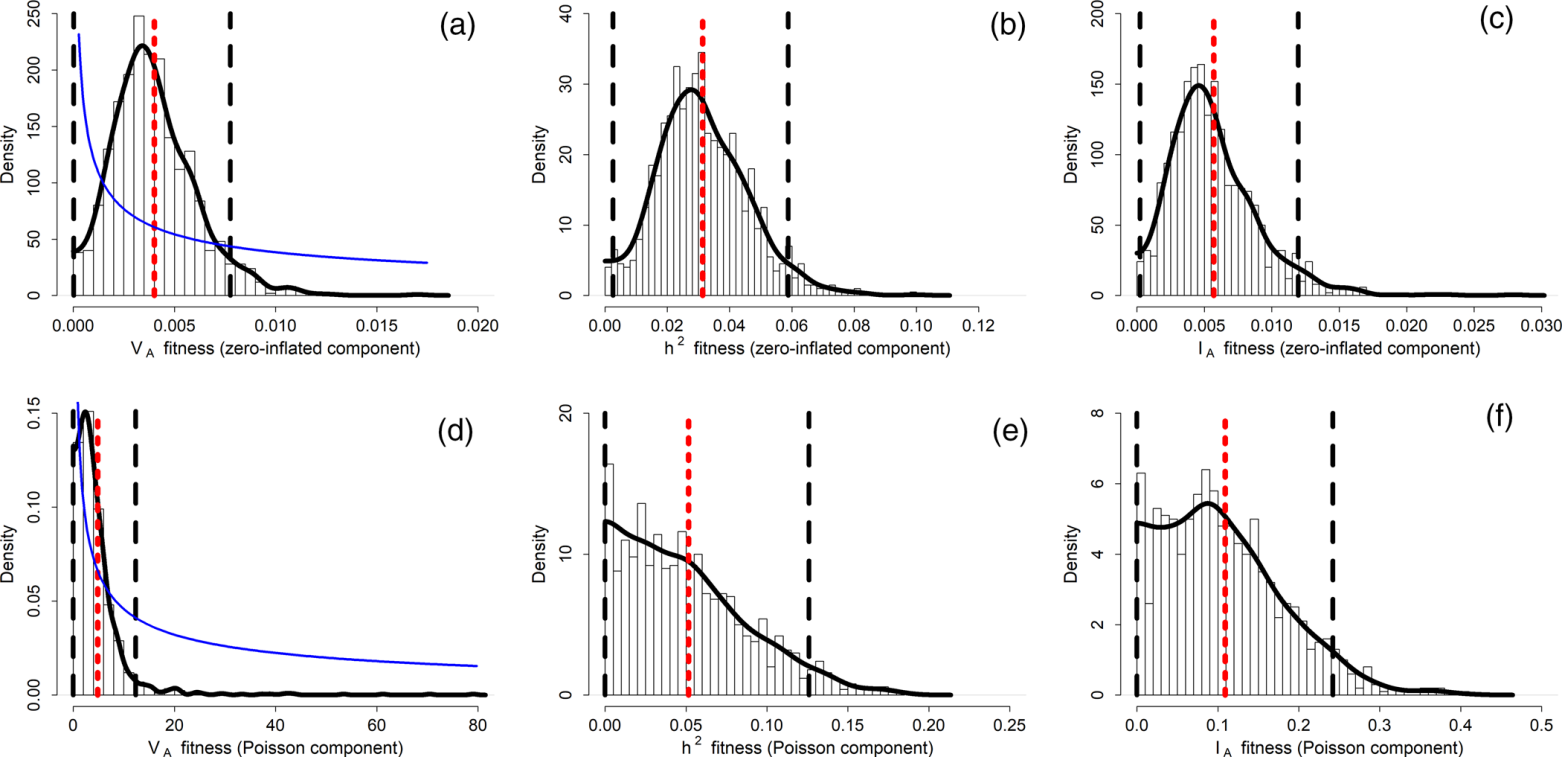
Posterior MCMC samples (bars), kernel density estimation (solid black line), posterior mean (red dotted line), 95% credible intervals (black dashed lines) and prior (solid blue line) for the (a) additive genetic variance (V_A_), (b) heritability (h^2^), and (b) evolvability (I_A_) of the zero–inflated component of lifetime fitness, and the (d) additive genetic variance (V_A_), (e) heritability (h^2^) and (f) evolvability (I_A_) of the Poisson component of lifetime fitness. Distributions are reported on the data scale.

Our quantitative genetic analysis of empirical data suggested that the additive genetic variance in the zero‐inflated component of lifetime fitness was not different from zero, as the posterior mode of the additive genetic variance was very close to, and the lower 95% CI limit leaning toward, zero (Table 1, Figure 2a‐c). Taken together, our combination of analyses of empirical and simulated data therefore suggested there to be low (lower than 0.05) to null additive genetic variance in the zero‐inflated component of lifetime fitness, but that we lack power to determine with higher precision whether such variance is effectively zero or nonzero but very small.

**Table 1:** Posterior modes and 95% credible intervals (in brackets) for data‐scale variance estimates from quantitative genetic analyses of lifetime fitness components

Fitness component	N_individuals_	Pop. Mean	V_P_	V_A_	h^2^	I_A_
zero‐inflated	5999	0.854 (0.777, 0.908)	0.119 (0.083, 0.173)	0.004 (0, 0.008)	0.031 (0.003, 0.059)	0.006 (0, 0.012)
Poisson	5999	5.71 (3.86, 10.2)	17.2 (20.4, 549)	2.29 (0.002, 12.3)	0.023 (0, 0.126)	0.088 (0, 0.242)

The results are shown for the zero‐inflated and Poisson components of the model. All statistics (Pop. Mean, population mean; V_P_, phenotypic variance; V_A_, additive genetic variance; h^2^, heritability; I_A_, evolvability) presented in the table are reported on the data‐scale.

The results for the Poisson component of lifetime fitness are less straightforward. Simulations showed that, given our data structure and pedigree, we would not be able to detect either *small but substantial* or larger signals for the Poisson component of fitness: we generated a Poisson component of fitness with a series of evolvability values (I_A_ = 0.00, 0.01, 0.05 and 0.10) and found that the lower 95% CI limit was on average zero in all cases (i.e., the lower 95% CI did not exceed a value of 0.0001 in the vast majority of the 100 replicates, Fig. S1‐4). Our analysis of the empirical data suggested that the additive genetic variance of the Poisson component did not differ from zero, given that the lower 95% CI limits of V_A_, h^2^ and I_A_ converged toward zero (Table 1, Figure 2d‐f). Altogether, the combination of empirical analyses and data simulations showed that we lacked power to determine where the additive genetic variance in the Poisson component of lifetime fitness falls within a rather large range of values (between “larger than 0.10” and zero).

Finally, simulation of a larger dataset with a deeper pedigree structure indicated that increasing our study to include four more generations of pedigreed individuals would lead to an important increase in statistical power, such that we would be able to detect additive genetic variances of at least 0.05 in both components of lifetime fitness. Estimated values of additive genetic variance were of similar magnitude to that of the simulated value (average posterior mode of 0.05 across the 100 replicates for both components of lifetime fitness), with nonzero lower 95% CI in both cases (95% CI = 0.031‐ 0.064 for the zero‐inflated component, and 95% CI = 0.009 −0.197 for the Poisson component, Fig. S5).

### QUANTITATIVE GENETICS OF ANNUAL FITNESS COMPONENTS

**Figure 3:**
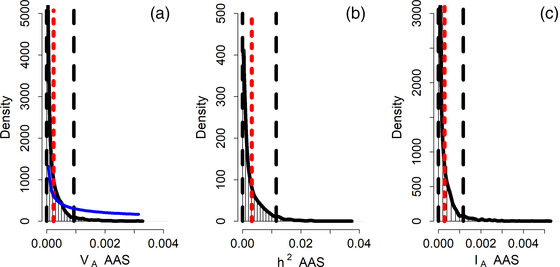
Posterior MCMC samples (bars), kernel density estimation (solid black line), posterior mean (red dotted line), 95% credible intervals (black dashed lines) and prior (solid blue line) for the (a) additive genetic variance (V_A_), (b) heritability (h^2^), and (c) evolvability (I_A_) of adult annual survival (AAS). Distributions are reported on the data scale.

**Figure 4:**
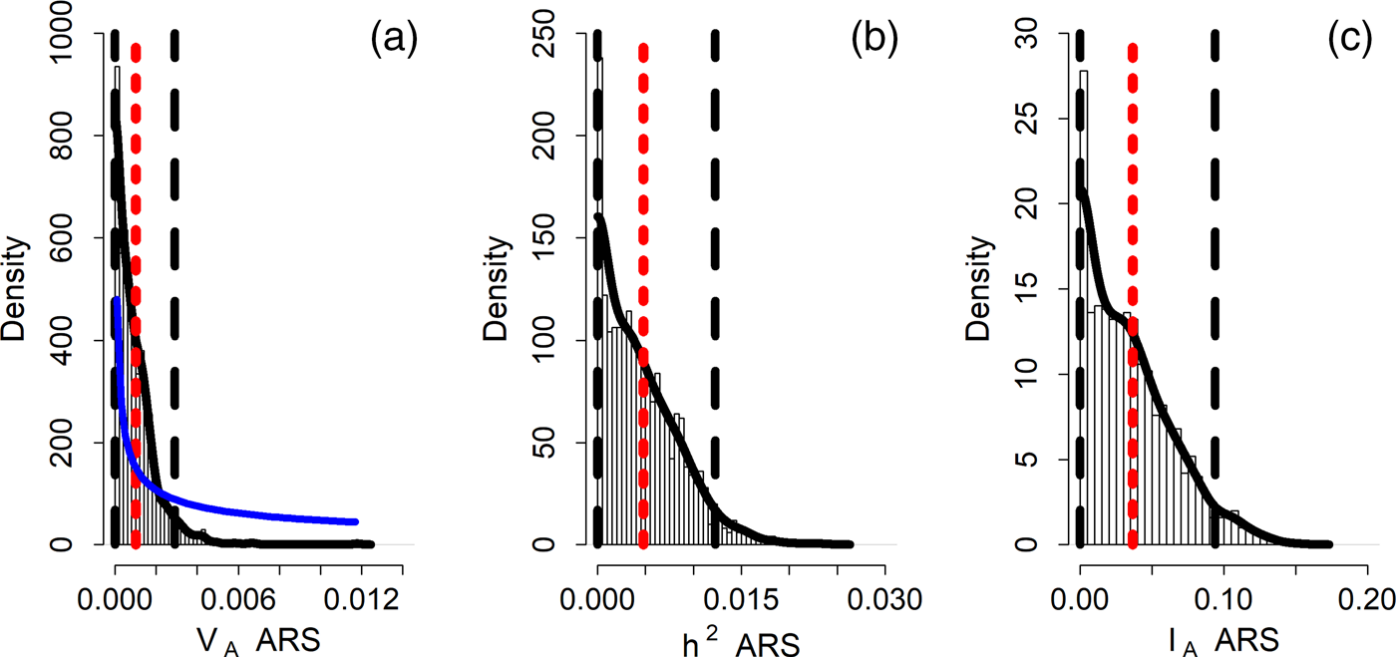
Posterior MCMC samples (bars), kernel density estimation (solid black line), posterior mean (red dotted line), 95% credible intervals (black dashed lines), and prior (solid blue line) for the (a) additive genetic variance (V_A_), (b) heritability (h^2^), and (c) evolvability (I_A_) of annual reproductive success (ARS). Distributions are reported on the data scale.

We investigated the annual reproductive success and adult annual survival of 836 fledglings that survived to adulthood and bred in our population (Table 2). The raw mean annual reproductive success was 0.70 ± 0.81 SD with a maximum of three fledglings (Fig. 1b). The posterior distribution of V_A_ for ARS converged toward zero (Table 2, Figure 4a‐c), suggesting that V_A_ is not different from zero. The raw mean adult annual survival probability was 0.85 ± 0.36 SD. The posterior modes of all quantitative genetic parameters for AAS were very close to zero (Table 2, Figure 3a‐c), with the lower 95% CI limit of all parameter estimates converging toward zero, again suggesting that V_A_ in AAS is not different from zero.

**Table 2:** Posterior modes and 95% credible intervals (in brackets) for data‐scale variance estimates from quantitative genetic analyses of annual reproductive success (ARS) and adult annual survival (AAS)

Fitness component	N_observations_	N_individuals_	Pop. Mean	V_P_	V_A_	h^2^	I_A_
ASS	6873	836	0.940 (0.855,0.972)	0.056 (0.029,0.126)	0.000 (0,0.001)	0.0001 (0,0.012)	0.000 (0,0.001)
ARS	6873	836	0.142 (0.108,0.236)	0.157 (0.115,0.365)	0.000 (0,0.003)	0.000 (0,0.012)	0.000 (0,0.094)

All statistics (Pop. Mean, population mean; V_P_, phenotypic variance; V_A_, additive genetic variance; h^2^, heritability; I_A_, evolvability) presented in the table are reported on the data scale.

## Discussion

The most direct measure of the adaptive potential of a population is its standing additive genetic variance in fitness (Fisher 1930). Here, we estimated additive genetic variances in lifetime and annual fitness components in a wild colony of common terns. On the one hand, our empirical findings indicated no evidence for substantial (or different from zero) additive genetic variance in lifetime fitness components, adult annual survival or annual reproductive success. On the other hand, data simulations demonstrated an overall lack of statistical power to detect *small but substantial* signals (i.e., V_A_ = 0.01), although statistical power differed between the two components of lifetime fitness: we would have power to detect slightly larger signals (additive genetic variances of at least 0.05) for the zero‐inflated, but not Poisson, component of fitness. As such, our work demonstrated that estimating additive genetic variance in fitness is very difficult in wild populations, partly due to the expected low values of genetic variation in fitness in locally adapted populations but also to the challenges associated with collecting sufficient phenotypic and pedigreed data.

### QUANTITATIVE GENETICS OF LIFETIME AND ANNUAL FITNESS COMPONENTS

There have been approximately 30 studies testing for additive genetic variance in fitness in the wild, with, to our knowledge, only four using non‐Gaussian animal models (McFarlane et al. 2014; McFarlane et al. 2015; Wolak et al. 2018; de Villemereuil et al. 2019). Our estimate of the additive genetic variance for the zero‐inflated component of lifetime fitness on the data‐scale was effectively zero, with a zero lower 95% CI limit (posterior mode V_A data‐scale_ = 0.004, 95% CI = 0 ‐ 0.008, Table 1), similar to results for another bird species, the hihi (posterior mode V_A data‐scale_ ∼ 0, 95% CI = 1.4×10^−11^ ‐ 0.0038, de Villemereuil et al. 2019). For the Poisson component, de Villemereuil et al. (2019) found a posterior mode of 0.0078 (95% CI = 2.3×10^−10^ ‐ 5.7). Our posterior mode estimate was overall larger (posterior mode V_A data‐scale_ = 2.29, Table 1) but associated with high uncertainty (95% CI = 0.002 ‐ 12.3), such that the estimates from both studies remain qualitatively similar. Given that our estimates of additive genetic variance in fitness showed very low or null values, our study implies that the adaptive potential of this natural population of common terns will be extremely limited, although the actual potential remains partially unknown, as our estimates were associated with high uncertainty. Moreover, it is important to note that we could only investigate the evolutionary potential of local recruits, as we did not have phenotypic and pedigree data to investigate the evolutionary potential of the total colony (i.e., local recruits and immigrants).

Additive genetic variance in lifetime fitness can theoretically be decomposed into additive genetic variances in its underlying components. The two primary components of our measure of lifetime fitness are juvenile survival and adult lifetime reproductive success. Our zero inflation in lifetime fitness is mainly due to low juvenile survival (i.e., 74% of fledglings did not locally return to the colony), while the Poisson process generating the observed fitness distribution mostly captures adult lifetime reproductive success. If we compare our nominally zero additive genetic variance in the zero‐inflated component of lifetime fitness (Table 1) with estimates from other studies that tested for additive genetic variance in juvenile survival, we observe some differences. For instance, the study of Wolak et al. (2018) on the song sparrow population of Mandarte Island reported evidence for nonzero V_A_ for juvenile survival.

Adult lifetime reproductive success is the sum of annual reproductive events across the life of an individual and hence can be decomposed into annual reproductive success and adult annual survival. Our quantitative genetic analyses of these two annual fitness components revealed a lack of substantial additive genetic variance for both (Table 2). This finding again contrasts with one from Mandarte's song sparrows, where there was evidence for moderate levels of additive genetic variance in ARS (especially for males) and close to zero in AAS, indicating that heritable ARS was the primary component of heritable adult lifetime reproductive success in that population (Wolak et al. 2018).

### LIMITATIONS OF STUDYING QUANTITATIVE GENETICS OF FITNESS IN THE WILD

Estimating quantitative genetic parameters with precision is a data‐hungry endeavor. Researchers are therefore faced with the challenge of collecting hard‐to‐quantify lifetime fitness data from an unbiased sample of the population (i.e., avoiding the “missing fraction” bias) that comprises a sufficiently large number of individuals of known relatedness (Burt 1995; Merilä and Sheldon 1999; Hendry et al. 2018). In addition, even when a large pedigree is available, additive genetic variance in fitness is often expected to be low, for instance, when populations are locally adapted, such that the power to detect small, close to zero, additive genetic variation in fitness may be low as well. As pointed out by Burt (1995), “it is very difficult to get an estimate that is statistically distinguishable from zero, and the sample sizes required to do so might easily lead to despair”. Our data simulations reveal that we would need at least four more generations of terns to significantly differentiate between an underpowered and a true zero estimate of additive genetic variance for the Poisson component of lifetime fitness. Increasing our pedigree by four more generations would require roughly two more decades of data collection, i.e., a nonnegligible amount of funding and logistical effort. This extrapolation should, however, be taken with care, as it is challenging to predict the population dynamics for the next twenty years and/or whether the relatedness structure of the population will increase or decrease as the rates of emigration and immigration may change with population growth (e.g., Szostek and Becker 2014). In light of the multiple constraints posed by data requirements and expected low values, negative results with respect to additive genetic variation in fitness should be discussed with caution. Nevertheless, simulations aimed at determining the statistical power of a given dataset and pedigree structure will help to distinguish a true negative result from a zero parameter estimated with high uncertainty (e.g., de Villemereuil et al. 2019).

In addition to the difficulty of estimating the heritability of fitness with precision, our knowledge of the genetic architecture of fitness components is limited. Extending our genomic understanding of fitness variation in wild populations will provide important insights into how genetic variation underpinning fitness may be maintained and, overall, will help to better predict the evolutionary dynamics of natural populations (Merilä and Sheldon 1999; Mackay 2001; Huang and Mackay 2016). Despite the clear benefits, genomic research based on quantitative trait loci (QTL) approaches or genome‐wide associations in natural populations is a challenge (Slate 2004; Slate et al. 2010; Jensen et al. 2014), partially due to the low power to detect QTLs, for instance, because studies suffer from low‐density linkage maps and/or relatively few genotyped individuals. Currently, the use of powerful next‐generation genomic techniques, however, allows for increased power in such studies.

A better understanding of the genetic architecture of fitness will also provide added benefits, as, for instance, it would allow a deeper understanding of the genetic underpinnings of complex traits such as fitness, which might be subjected to different pleiotropic effects (Mackay 2001). For instance, antagonistic pleiotropy is often assumed to underlie the negative phenotypic correlation between the two main components of lifetime fitness: survival and reproductive success (also observed in the terns: Vedder et al. 2021).

## Conclusion

Our quantitative genetic study of fitness in a wild population of common terns reported low to zero estimates of additive genetic variance in lifetime and annual fitness components, which were associated with high uncertainty. Those analyses, however, were overshadowed by a lack of statistical power to detect additive genetic variation in fitness more accurately and precisely. The continuation of long‐term individual‐based studies should be safeguarded (also see Clutton‐Brock and Sheldon 2010), such that the maturation of long‐term studies will offer improved opportunities for testing genetic variation in natural populations, which, thanks to the recent development of appropriate statistical and theoretical frameworks (de Villemereuil et al. 2016; Bonnet et al. 2019; Morrissey and Bonnet 2019), will help to improve our understanding of the genetics of fitness in the wild. Ultimately, a robust quantification of the standing additive genetic variation in fitness will inform us about the rate of adaptation of populations and allow a better understanding of their viability in the face of the deleterious environmental effects resulting from current climate and global changes.

## AUTHOR CONTRIBUTIONS

M.M. conceived the study with input from S.B. and A.C. M.M. designed and conducted the analyses and wrote the manuscript. S.B. manages the tern data and collated the dataset. All authors contributed to editing the final paper.

## CONFLICT OF INTEREST

The authors declare no conflict of interest.

## DATA ARCHIVING

Data have been archived in the Dryad Digital Repository: https://doi.org/10.5061/dryad.8kprr4xqj (Moiron et al. 2022).

Associate Editor: G. Marroig

Handling Editor: A. G. McAdam

## Supporting information

Supplementary Table S1. Model of lifetime fitness with parental effects.
Supplementary Table S2. Model of lifetime fitness with brood effects.
Supplementary Table S3. Models of AAS and ARS without age.
Supplementary Text S1. Pedigree information.
Supplementary Text S2. Prior specifications.
Supplementary Text S3 and Figs. S1‐S4. Data simulations.
Supplementary Text S4 and Fig. S5 Data simulations with increased pedigree depth.
Supplementary Fig. S6. Temporal variation in population size
