# Supplementary material for: The quantitative genetics of fitness in a wild seabird
Source: Evolution. 2022 Jun 15;76(7):1443–52. doi: 10.1111/evo.14516 (PMC9544722; doi:10.1111/evo.14516)
Supplement: Supplementary file 1 — Supplementary Table S1. Model of lifetime fitness with parental effects. Supplementary Table S2. Model of lifetime fitness with brood effects. Supplementary Table S3. Models of AAS and ARS without age. Supplementary Text S1. Pedigree information. Supplementary Text S2. Prior specifications. Supplementary Text S3 and Figs. S1‐S4. Data simulations. Supplementary Text S4 and Fig. S5 Data simulations with increased pedigree depth. Supplementary Fig. S6. Temporal variation in population size [file EVO-76-1443-s001.docx]

**The quantitative genetics of fitness in a wild seabird**

**—Online Supplementary Material—**

Supplementary Table S1. Model of lifetime fitness with parental effects Page 2

Supplementary Table S2. Model of lifetime fitness with brood effects Page 4

Supplementary Table S3. Models of AAS and ARS without age Page 6

Supplementary Text S1. Pedigree information Page 7

Supplementary Text S2. Prior specifications Page 8

Supplementary Text S3 and Figs. S1-S4. Data simulations Page 9

Supplementary Text S4 and Fig. S5 Data simulations with increased pedigree depth Page 15

Supplementary Fig. S6. Temporal variation in population size Page 18

**SUPPLEMENTARY TABLE S1. MODEL OF LIFETIME FITNESS WITH PARENTAL EFFECTS**

**Table S1A.** Posterior modes and 95% Credible Intervals (in brackets) for latent-scale variance estimates from quantitative genetic analyses of lifetime fitness components including maternal and paternal effects.

| **Fitness**  **component** | **N_individuals_** | **V_A_** | **V_M_** | **V_F_** | **V_YEAR_** | **V_E_** |
| --- | --- | --- | --- | --- | --- | --- |
| Zero-  inflated | 1271 | 0.004 (0,0.89) | 0.003 (0,0.551) | 0.004 (0,0.621) | 2.891 (1.528,8.102) | 1  (1,1) |
| Poisson |  | 0.002 (0,0.344) | 0.001 (0,0.155) | 0.001 (0,0.148) | 0.000 (0,0.159) | 0.403 (0.206,0.728) |

Results are shown for the Zero-inflated and Poisson components of the model. All statistics (V_A_, additive genetic variance; V_M_, maternal variance; V_F,_ paternal variance; V_E_, residual variance) presented in the table are reported on the latent scale.

**Table S1B.** Posterior modes and 95% Credible Intervals (in brackets) for data-scale variance estimates from quantitative genetic analyses of lifetime fitness components including maternal and paternal effects.

| **Fitness component** | **N_individuals_** | **Pop. Mean** | **V_P_** | **V_A_** | **V_M_** | **V_F_** | **h^2^** | **I_A_** |
| --- | --- | --- | --- | --- | --- | --- | --- | --- |
| Zero- inflated | 1271 | 0.858 (0.754,0.916) | 0.122 (0.08,0.189) | 0.000 (0,0.005) | 0.000 (0,0.004) | 0.000 (0,0.004) | 0.000 (0,0.038) | 0.000 (0,0.008) |
| Poisson |  | 5.838 (4.149,8.525) | 35.676 (17.076,85.362) | 0.101 (0,14.429) | 0.030 (0,6.231) | 0.045 (0,6.479) | 0.002 (0,0.264) | 0.002 (0,0.344) |

Results are shown for the Zero-inflated and Poisson components of the model. All statistics (Pop. Mean, population mean; V_P_, phenotypic variance; V_A_, additive genetic variance; V_M_, maternal variance; V_F,_ paternal variance; h^2^, heritability; I_A_, evolvability) presented in the table are reported on the data-scale.

**SUPPLEMENTARY TABLE S2. MODEL OF LIFETIME FITNESS WITH BROOD EFFECTS**

**Table S2A.** Posterior modes and 95% Credible Intervals (in brackets) for latent-scale variance estimates from quantitative genetic analyses of lifetime fitness components including brood effects.

| **Fitness component** | **N_individuals_** | **V_A_** | **V_BROOD_** | **V_YEAR_** | **V_E_** |
| --- | --- | --- | --- | --- | --- |
| Zero-inflated | 5995 | 0.825 (0.083,2.225) | 1.135 (0,2.174) | 3.250 (1.324,7.623) | 1  (1,1) |
| Poisson |  | 0.001 (0,0.18) | 0.000 (0,0.088) | 0.388 (0.127,1.175) | 0.375 (0.248,0.539) |

Results are shown for the Zero-inflated and Poisson components of the model. All statistics (V_A_, additive genetic variance; V_BROOD_, brood variance; V_YEAR,_ year variance; V_E_, residual variance) presented in the table are reported on the latent scale.

**Table S2B.** Posterior modes and 95% Credible Intervals (in brackets) for data-scale variance estimates from quantitative genetic analyses of lifetime fitness components including brood effects.

| **Fitness component** | **N_individuals_** | **Pop. Mean** | **V_P_** | **V_A_** | **V_BROOD_** | **h^2^** | **I_A_** |
| --- | --- | --- | --- | --- | --- | --- | --- |
| Zero-inflated | 5995 | 0.870 (0.786,0.914) | 0.113 (0.079,0.168) | 0.005 (0.001,0.009) | 0.005 (0,0.01) | 0.041 (0.009,0.072) | 0.005 (0.001,0.014) |
| Poisson |  | 6.594 (4.626,10.493) | 65.627 (25.811,377.232) | 0.151 (0,10.251) | 0.015 (0,5.2) | 0.001 (0,0.1) | 0.001 (0,0.18) |

Results are shown for the Zero-inflated and Poisson components of the model. All statistics (Pop. Mean, population mean; V_P_, phenotypic variance; V_A_, additive genetic variance; V_BROOD_, brood variance; h^2^, heritability; I_A_, evolvability) presented in the table are reported on the data-scale.

**SUPPLEMENTARY TABLE S3. MODELS OF AAS AND ARS WITHOUT AGE**

**Table S3.** Posterior modes and 95% Credible Intervals (in brackets) for data-scale variance estimates from quantitative genetic analyses of annual reproductive success (ARS) and adult annual survival (AAS) not including age as a linear predictor.

| **Fitness component** | **N_observations_** | **N_individu_als** | **Pop. Mean** | **V_P_** | **V_A_** | **h^2^** | **I_A_** |
| --- | --- | --- | --- | --- | --- | --- | --- |
| ASS | 6873 | 836 | 0.856 (0.746,0.932) | 0.124  (0.065494,0.191) | 0.000 (0,0.000) | 0.000 (0,0.002) | 0.000 (0,0.000) |
| ARS |  |  | 0.509 (0.245,1.456) | 0.123  (0.436,80.462) | 0.000 (0,0.034) | 0.000 (0,0.005) | 0.000 (0,0.046) |

All statistics (Pop. Mean, population mean; V_P_, phenotypic variance; V_A_, additive genetic variance; h^2^, heritability; I_A_, evolvability) presented in the table are reported on the data scale.

## SUPPLEMENTARY TEXT S1. PEDIGREE

A social pedigree was constructed from the observations of parents and their fledged offspring. Due to the very low levels of extra-pair paternity in the population (97.1% of true siblings found among 22 broods, González-Solís et al. 2001), the social pedigree is a good approximation of the genetic pedigree. For the period 1992-2019, the pruned pedigree comprised 6290 individuals. The maximum depth was five generations, the number of paternities and maternities 2417 and 2520, respectively. The numbers of full, paternal and maternal siblingships were 2594, 10229 and 9807, respectively. The histogram below represents the relatedness between pairs of individuals present in the pruned pedigree for this population of common terns.


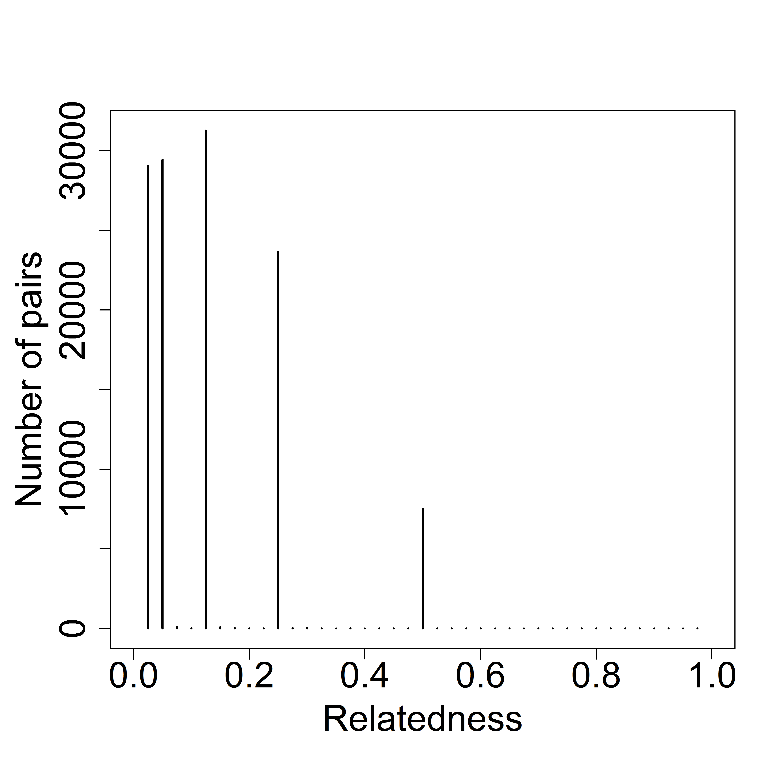


**SUPPLEMENTARY TEXT S2. PRIOR SPECIFICATION**

We used different prior specifications for the different quantitative genetic parameters reported in the main text (see below). We also ran a prior sensitivity analysis and found that the reported parameters were not substantially influenced by the prior chosen, since posterior modes and 95% CI were similar (Results not shown). We therefore concluded that our results and interpretations were robust to reasonable alternative priors.

**Priors used in the analysis for lifetime fitness components:**

G_A_: V = diag(2)/2, nu = 2, alpha.mu = c(0,0), alpha.V = diag(2)*1000)

G_C_: V = diag(2)/2, nu = 2, alpha.mu = c(0,0), alpha.V = diag(2)*1000)

R: V = diag(2), nu = 1, fix = 2

**Priors used in the analysis for Adult Reproductive Success:**

G_A_: V = 1*0.02, nu = 1, alpha.mu =0, alpha.V = 1000

G_PE_: V = 1, nu = 1, alpha.mu =0, alpha.V = 1000

G_YEAR_: V = 1*0.02, nu = 1, alpha.mu =0, alpha.V = 1000

R: V = 1, nu= 1

**Priors used in the analysis for Adult Annual Survival:**

G_A_: V = 1, nu = 1, alpha.mu =0, alpha.V = 10

G_PE_: V = 1, nu = 1, alpha.mu =0, alpha.V = 10

G_YEAR_: V = 1, nu = 1, alpha.mu =0, alpha.V = 10

R: V = 1, fix= 1

**SUPPLEMENTARY TEXT S3. DATA SIMULATIONS**

We performed a data simulation analysis to investigate whether we can effectively detect *small, but substantial* additive genetic variance (*sensu* de Villemereuil et al. 2019) in lifetime fitness components given our sample size and pedigree structure.

**Methods:** We used the pedigree and data structure of the common tern population at the Banter See to simulate a zero-inflated Poisson lifetime fitness trait, and compared the results of the quantitative genetic model with those resulting from the model applied to our empirical data. Following de Villemereuil et al. (2019), we used a data-scale additive genetic variance of fitness of 0.01 for the Poisson component. Unlike de Villemereuil et al. (2019), we also used an evolvability of 0.01 for the Zero-inflated component. We chose a value of 0.01 because values below that arbitrary threshold would be considered inconsequential (de Villemereuil et al. 2019). Besides generating additive genetic variances, we simulated the effects of hatch-year (i.e., cohort) by adding it as a random effect. Residual variance for the Zero-inflated part of the simulated fitness trait was fixed to one. This way, we followed the structure of our fitted Zero-inflated Poisson model and accounted for the precision lost by adding random effects. The simulation analysis consisted of 100 replicates and was performed in the R-package *MCMCglmm* (Hadfield 2010). The R-code for the data simulations was adapted from that of de Villemereuil et al. (2019) and is available on GitHub (https://github.com/MariaMoiron/Va-of-fitness).

**Results:** Our data simulations indicated that, given our data structure and pedigree, we would not be able to detect *small but substantial* additive genetic variance for either of the two components of lifetime fitness (Figs. S1). The average posterior modes of data-scale additive genetic variance of fitness across the 100 replicates were 0.012 (with associated 95% CI = 0 – 0.023) for the Zero-Inflated component, and 0.028 (with associated 95% CI = 0 – 0.020) for the Poisson component. For both components, the lower 95% limit was effectively zero in most of the replicates (i.e., did not exceed a value of 0.0001, Fig. S1). Given that a scenario without simulated additive genetic variance (i.e., I_A_ = 0.00) could give rise to a similar pattern, we ran the same data simulation as described above, but modifying the values of I_A_ so that instead of simulating a value of I_A_ = 0.01, we now simulated an effectively zero value. As expected, we found a similar pattern of the average posterior mode for both components being close to zero (average of ~ 0.001 across simulations). Again, the lower 95% CI did not exceed a value of 0.0001 in most of replicates (Fig. S2). As a final step, we simulated two other values of additive genetic variance of fitness that were substantially larger: I_A_ = 0.05 and 0.1. On the one hand, the data simulation analysis showed that we had sufficient power to detect larger values of additive genetic variance for the Zero-inflated component. We found that estimated values of genetic variance were of similar magnitude to that of the simulated value (average posterior mode of 0.05 across the 100 replicates for the simulations of 0.05, and 0.1 for the simulations of 0.1), with the lower 95% CI exceeding a value of 0.0001 in all cases (Figs. S3-4). On the other hand, the data simulation analysis showed that we did not have sufficient power to detect larger values of additive genetic variance (i.e., 0.05 or 0.1) for the Poisson component of lifetime fitness. We found an average posterior mode of 0.039 across the 100 replicates for the simulations of 0.05, and 0.09 for the simulations of 0.1, with the lower 95% CI leaning towards zero in both cases (i.e., lower 95% CI only exceeded a value of 0.0001 in 27% and 42% of the cases, respectively Fig. S3-4).

**Figure S1.** Data simulation analysis of lifetime fitness components. Left and right panels show the posterior estimates for all 100 replicates of data-scale additive genetic variance of the Zero-inflated component, and of the Poisson component of simulated lifetime fitness, respectively. Posterior modes are represented as red dots and 95% credible intervals as grey horizontal lines. The black vertical line is the simulated value (i.e., a value of 0.01).


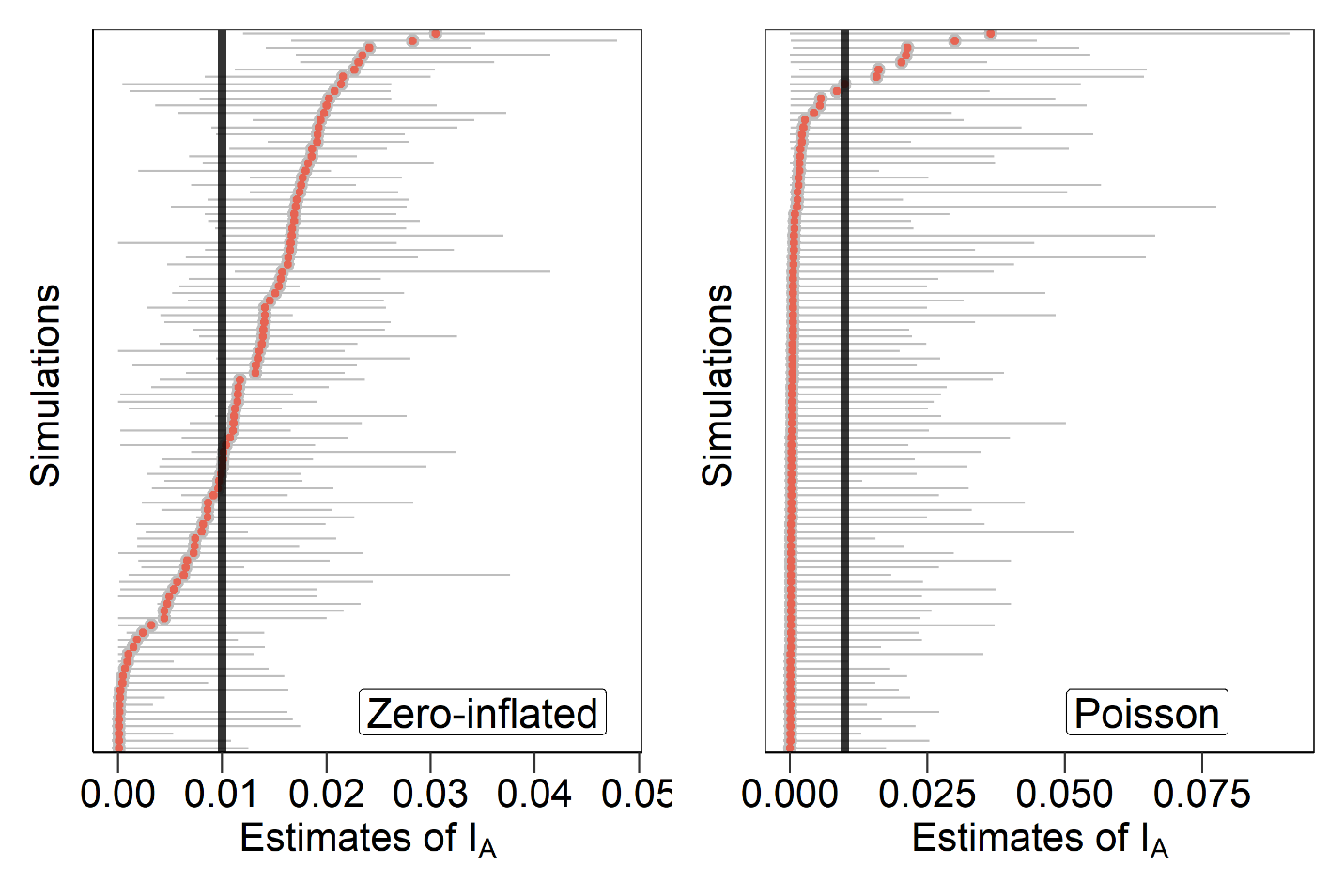


**Figure S2.** Data simulation analysis of lifetime fitness components. Left and right panels show the posterior estimates for all 100 replicates of data-scale additive genetic variance of the Zero-inflated component, and of the Poisson component of simulated lifetime fitness, respectively. Posterior modes are represented as red dots and 95% credible intervals as grey horizontal lines. The black vertical line is the simulated value (i.e., a value of ~0.00).


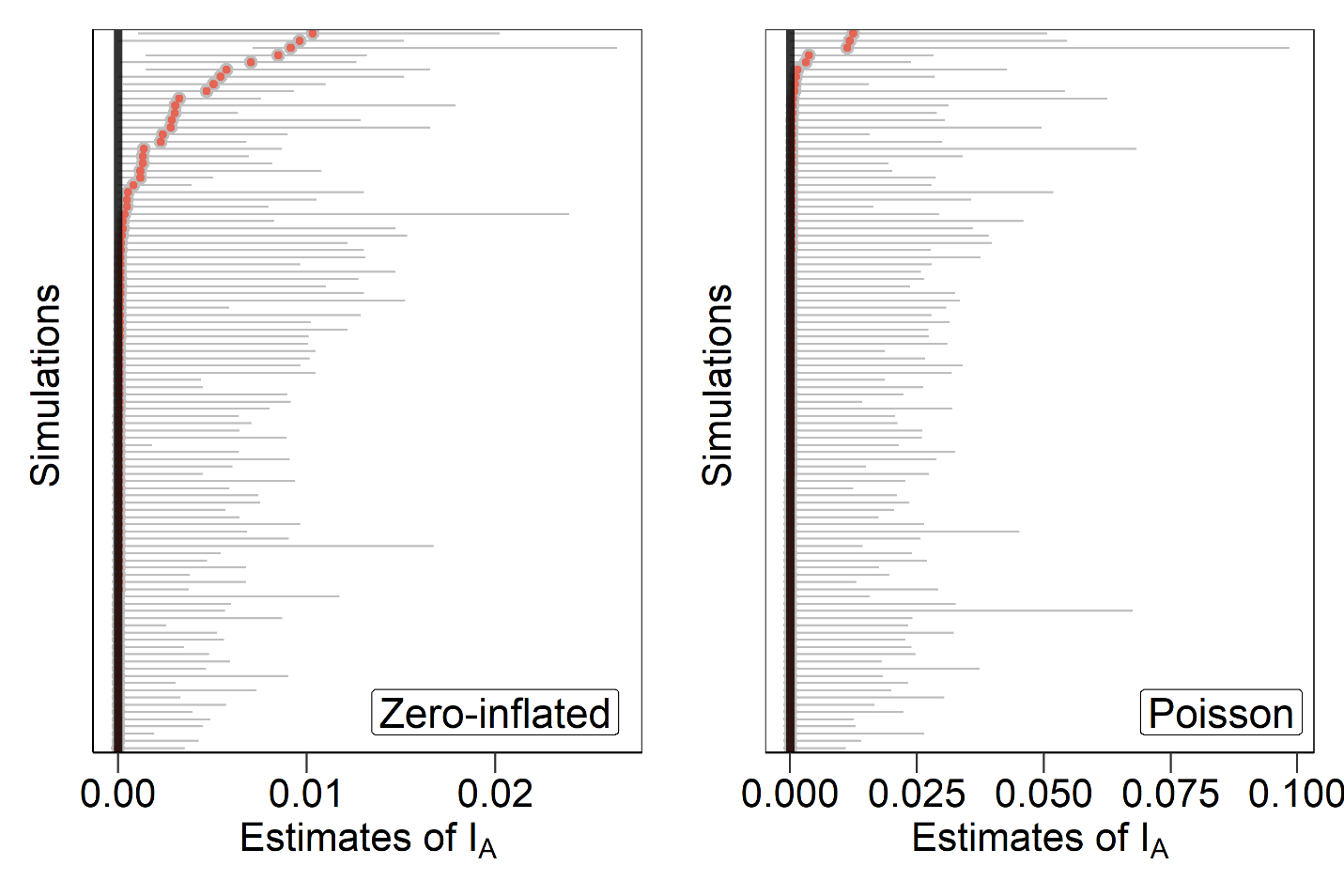


**Figure S3.** Data simulation analysis of lifetime fitness components. Left and right panels show the posterior estimates for all 100 replicates of data-scale additive genetic variance of the Zero-inflated component, and of the Poisson component of simulated lifetime fitness, respectively. Posterior modes are represented as red dots and 95% credible intervals as grey horizontal lines. The black vertical line is the simulated value (i.e., a value of 0.05).


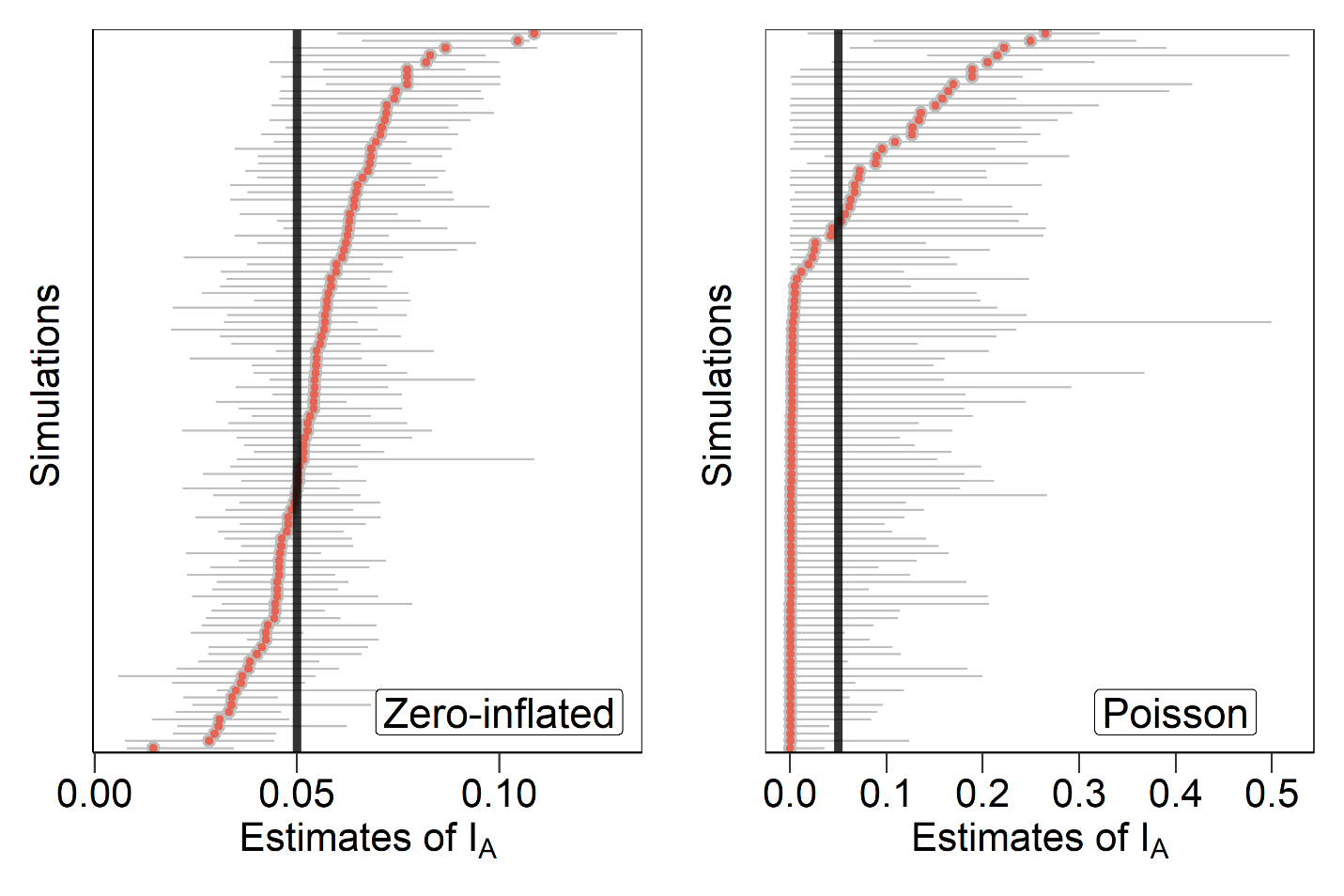


**Figure S4.** Data simulation analysis of lifetime fitness components. Left and right panels show the posterior estimates for all 100 replicates of data-scale additive genetic variance of the Zero-inflated component, and of the Poisson component of simulated lifetime fitness, respectively. Posterior modes are represented as red dots and 95% credible intervals as grey horizontal lines. The black vertical line is the simulated value (i.e., a value of 0.1).


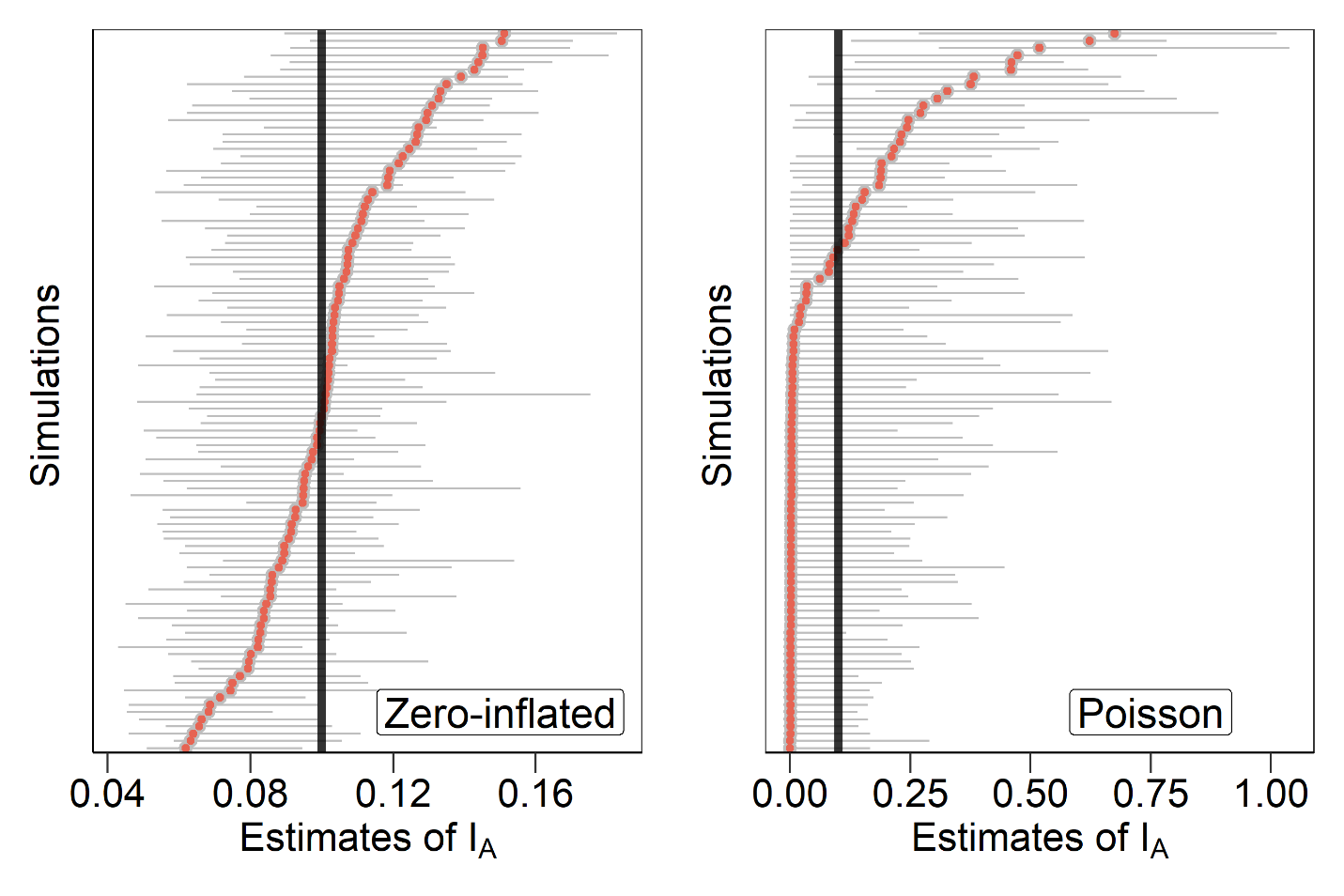


**SUPPLEMENTARY TEXT S4. DATA SIMULATIONS WITH INCREASED PEDIGREE DEPTH**

To test how the accretion of a longer dataset, consisting of a larger number of individuals and a pedigree containing a larger number of generations, might improve our power to detect small levels of additive genetic variance in lifetime fitness (I_A_ = 0.05), we simulated a new phenotypic dataset and pedigree that included four more generations.

Because the structure of the new pedigree will likely influence the outcome of the simulations, we first simulated a pedigree that resembled our empirically-obtained pedigree. To do so, we simulated 1060 individuals and 530 breeding pairs over five generations, where each pair contributed 4 offspring to the next generation. Simulations were set up with 600 immigrants per generation. This new pedigree comprised 6360 records, with 2900 paternities and maternities each. The numbers of full, maternal and paternal siblingships were 1389, 3395 and 3037, respectively, i.e. of similar magnitude as in our empirical pedigree, although it had lower mean siblingship size. We ran a sensitivity test to assess how our parameter choice might affect the outcome of the analyses. We tried different parameters in terms of number of breeding pairs and immigrants, and found that despite those parameter changes, the results were all similar among them and to our main data simulations (i.e., we would be able to detect I_A_=0.05 for the Zero-inflated but not I_A_=0.1 for Poisson component of fitness), suggesting that our approach was robust. We then simulated another pedigree, using the same parameters as before but with four more generations than the actual pedigree. We simulated the pedigree using the R-package *nadiv* (Wolak 2012), and the phenotypes using the same approach as in the main data simulations. We then fitted the same statistical model as in the main analysis to this new dataset.

We found that by adding four more generations of individuals to our simulated pedigree, we would have sufficient statistical power to detect relatively small additive genetic variances (V_A_= 0.05) for both components of lifetime fitness: estimated values of genetic variance were of similar magnitude to that of the simulated value (average posterior mode of ~0.05 across the 100 replicates for both components of lifetime fitness), with the lower 95% CI limit being non-zero in both cases (95% CI =0.032- 0.065 and lower 95% CI exceeded a value of 0.0001 in 100% of replicates for Zero-Inflated component, and 95% CI = 0.021 -0.151 for Poisson component and 40% of the lower 95% CI exceeded a value of 0.0001, Fig. S5).

**Figure S5.** Data simulation analysis of lifetime fitness components using a pedigree that is four more generations deep. Left and right panels show the posterior estimates for all 100 replicates of data-scale additive genetic variance of the Zero-inflated component, and of the Poisson component of simulated lifetime fitness, respectively. Posterior modes are represented as red dots and 95% credible intervals as grey horizontal lines. The black vertical line is the simulated value (i.e., a value of 0.05).

**
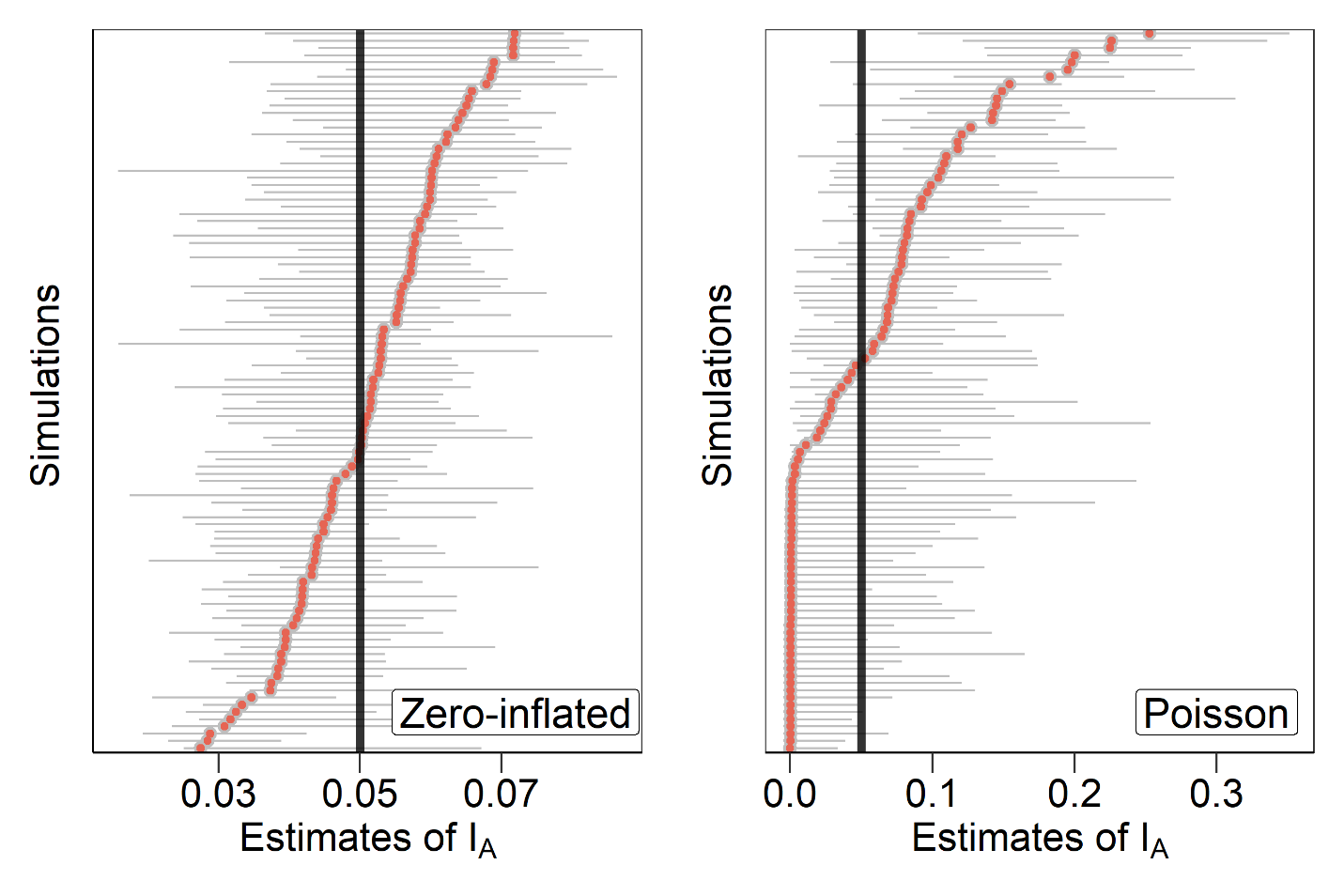
**

**Supplementary Fig. S6. Temporal variation in population size**

**Figure S6.** Variation in population size, assessed as the total number of breeding pairs, across the 28-year study period. Black dots represent annual estimates; grey lines connect these values.


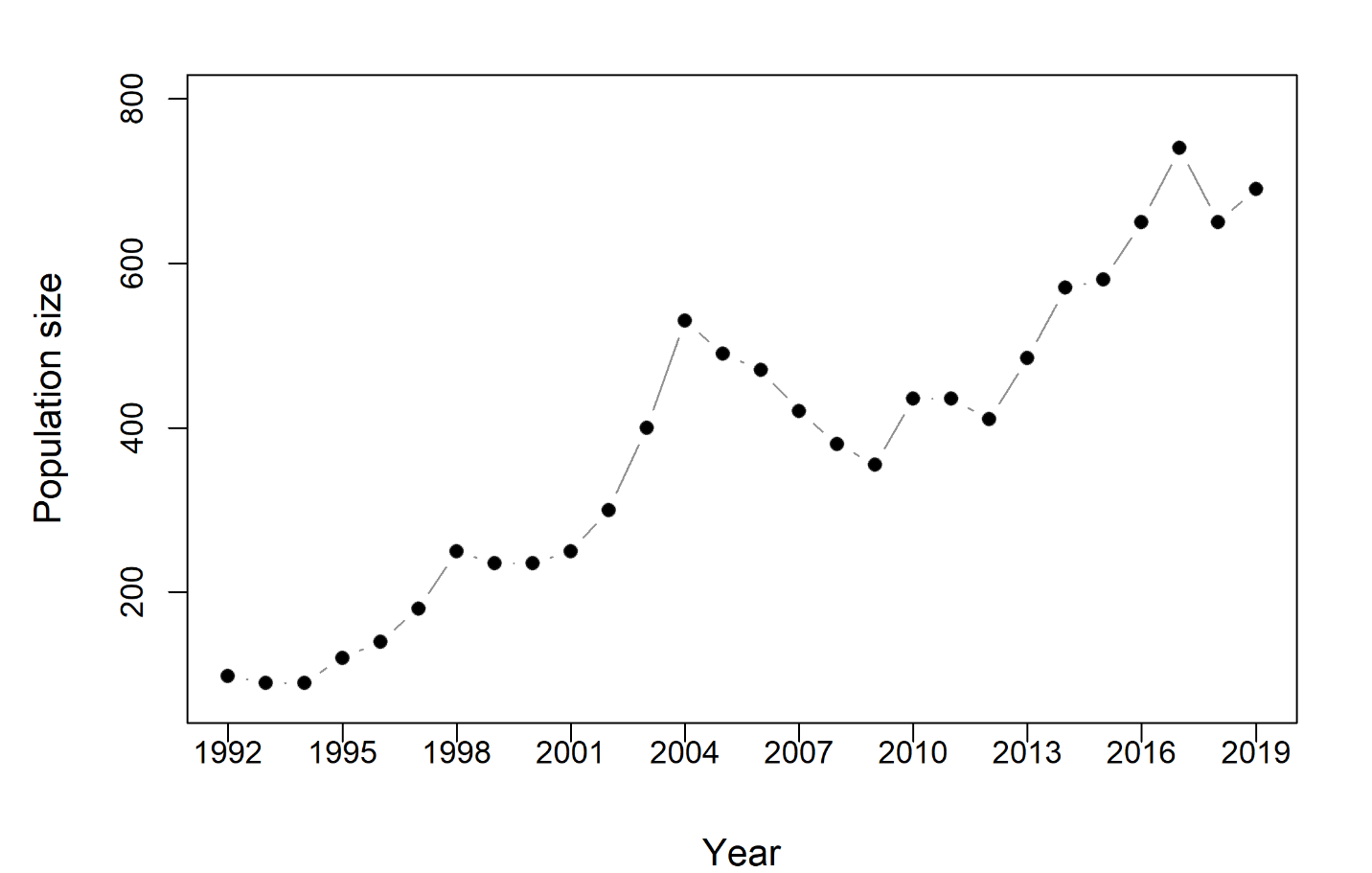


**REFERENCES**

de Villemereuil, P., A. Rutschmann, K. D. Lee, J. G. Ewen, P. Brekke, and A. W. Santure. 2019. Little Adaptive Potential in a Threatened Passerine Bird. Curr. Biol. 29:889-894.e3.

Hadfield, J. 2010. MCMC Methods for Multi-response Generalized Linear Mixed Models : The MCMCglmm R Package. J. Stat. Softw. 33:1–22.

Wolak, M.E. 2012. “nadiv: an R package to create relatedness matrices for estimating non-additive genetic variances in animal models.” Methods in Ecology and Evolution, 3(5), 792-796.
